# Bispecific GRPR-Antagonistic Anti-PSMA/GRPR Heterodimer for PET and SPECT Diagnostic Imaging of Prostate Cancer

**DOI:** 10.3390/cancers11091371

**Published:** 2019-09-14

**Authors:** Bogdan Mitran, Zohreh Varasteh, Ayman Abouzayed, Sara S. Rinne, Emmi Puuvuori, Maria De Rosa, Mats Larhed, Vladimir Tolmachev, Anna Orlova, Ulrika Rosenström

**Affiliations:** 1 Department of Medicinal Chemistry, Uppsala University, 751 23 Uppsala, Sweden; bogdan.mitran@ilk.uu.se (B.M.); zohreh.varasteh@tum.de (Z.V.); ayman.abouzayed@ilk.uu.se (A.A.); sara.rinne@ilk.uu.se (S.S.R.); mats.larhed@ilk.uu.se (M.L.); ulrika.rosenstrom@ilk.uu.se (U.R.); 2 Department of Nuclear Medicine, Klinikum rechts der Isar der TUM, 81675 Munich, Germany; 3 Drug Discovery Unit, Ri.MED Foundation, 90133 Palermo, Italy; 4 Science for Life Laboratory, Department of Medicinal Chemistry, Uppsala University, 751 23 Uppsala, Sweden; 5 Department of Immunology, Genetics and Pathology, Uppsala University, 751 23 Uppsala, Sweden; vladimir.tolmachev@igp.uu.se

**Keywords:** PSMA, GRPR, heterodimer, molecular imaging, prostate cancer, PET, SPECT

## Abstract

Simultaneous targeting of the prostate-specific membrane antigen (PSMA) and gastrin-releasing peptide receptor (GRPR) could improve the diagnostic accuracy in prostate cancer (PCa). The aim of this study was to develop a PSMA/GRPR-targeting bispecific heterodimer for SPECT and positron emission tomography (PET) diagnostic imaging of PCa. The heterodimer NOTA-DUPA-RM26 was produced by manual solid-phase peptide synthesis. NOTA-DUPA-RM26 was labeled with ^111^In and ^68^Ga, with yields >98%, and demonstrated a high stability and binding specificity to PSMA and GRPR. IC_50_ values for ^nat^In-NOTA-DUPA-RM26 were 4 ± 1 nM towards GRPR and 824 ± 230 nM towards PSMA. An in vivo binding specificity 1 h pi of ^111^In-NOTA-DUPA-RM26 in PC3-PIP-xenografted mice demonstrated partially blockable tumor uptake when co-injected with an excess of PSMA- or GRPR-targeting agents. Simultaneous co-injection of both agents induced pronounced blocking. The biodistribution of ^111^In-NOTA-DUPA-RM26 and ^68^Ga-NOTA-DUPA-RM26 revealed fast activity clearance from the blood and normal organs via the kidneys. Tumor uptake exceeded normal organ uptake for both analogs 1 h pi. ^68^Ga-NOTA-DUPA-RM26 had a significantly lower tumor uptake (8 ± 2%ID/g) compared to ^111^In-NOTA-DUPA-RM26 (12 ± 2%ID/g) 1 h pi. Tumor-to-organ ratios increased 3 h pi, but decreased 24 h pi, for ^111^In-NOTA-DUPA-RM26. MicroPET/CT and microSPECT/CT scans confirmed biodistribution data, suggesting that ^68^Ga-NOTA-DUPA-RM26 and ^111^In-NOTA-DUPA-RM26 are suitable candidates for the imaging of GRPR and PSMA expression in PCa shortly after administration.

## 1. Introduction

Until recently, the diagnosis of prostate cancer (PCa) was based on measuring the concentration of prostate-specific antigen (PSA) in blood, a pathological examination of biopsy material, positron emission tomography (PET) imaging of metabolic activity using ^18^F-flourodeoxyglucose (FDG) or ^11^C-acetate, and proliferation activity using radiolabeled choline [1,2]. Bombesin agonistic analogues for the imaging of gastrin-releasing peptide receptor (GRPR) expression were also proposed, but their clinical implementation was limited due to their strong physiological action [3]. 

The development of small-molecular-weight prostate-specific membrane antigen (PSMA)-targeting derivatives of urea was a big breakthrough in the diagnosis of PCa [4]. Challenging clinically relevant diagnostic questions related to, e.g., accurate primary staging and restaging after biochemical recurrence, guided biopsy, and treatment monitoring, might be solved for many PCa patients in the case of the regulatory approval of PSMA-targeting imaging agents. Dozens of Glu-Ureido derivatives have been clinically tested for PET and SPECT imaging modalities. Among them, the most frequently used is ^68^Ga-labeled PSMA-11 (derivative containing the HBED-CC chelator) [5].

Despite the apparent success, the imaging of PCa lesions requires further improvement. A meta-analysis of published data concerning PSMA imaging for 1256 patients [6] reported a 70% sensitivity in the detection of intraprostatic cancer lesions and a 61% sensitivity in the detection of lymph node lesions. In fairness, it should be mentioned that the sensitivity in detecting PCa lesions is 39–42% for CT/MR [7], 37% for FDG-PET [8], and 50% for choline PET [9]. Importantly, the treatment and management of PCa patients is highly dependent on the results obtained from staging and re-staging; local and systemic diseases requiring different therapies. Changes in therapeutic management after PSMA-targeted imaging were reported for up to 75% of PCa patients [4]. 

The main reasons for the relatively low sensitivity of PSMA-targeted imaging of PCa are (i) the absence of or insufficient PSMA expression in about 10% of primary PCa, (ii) presence of lesions with downregulated PSMA due to therapy resistance (e.g., neuroendocrine-differentiated PCa), (iii) difficulties in the detection of lesions below 5 mm (80% of lymph node metastases are smaller than 8 mm), and (iv) lesion location in the proximity of the urinary bladder [4,10]. Consequently, new approaches should be considered to improve the diagnostic accuracy.

Another relevant target for the diagnostic imaging of PCa is GRPR, which is expressed in earlier stages of PCa [11]. The gallium-68-labeled GRPR antagonist RM2 was used in several clinical studies, with promising results [12,13,14]. A small clinical study (*n* = 14) showed a high imaging sensitivity of 88% in PCa [13]. The direct comparison of PSMA- and GRPR-targeting imaging agents in PCa patients (*n* = 7) demonstrated that both agents accumulate similarly in the identified lesions (with a few exceptions when only one of the agents had a high uptake) and the authors concluded that some patients should benefit from imaging with both agents [15].

Generally, the imaging sensitivity can be improved by increasing tumor-to-background ratios [16]. Several approaches can be used for this, e.g., the use of agents with a higher affinity and/or faster blood clearance, optimization of the injected dose of targeting agent, decreasing the unspecific activity uptake in background tissues, or increasing the avidity of the agent. The avidity of the imaging probe can be increased by simple multimerization of the agent, but in the case of PCa, this would hardly solve the problem of heterogeneity of PSMA and GRPR expression. A heterodimer combining PSMA and GRPR binding moieties might be an attractive option.

Recently, three groups reported the development of PSMA/GRPR-targeting heterodimers, all using a urea-based PSMA inhibitor and a bombesin-derived GRPR agonist [17,18,19]. Heterodimers were labeled with positron-emitting radiometals and demonstrated that they could bind to both molecular targets. However, the choice of a GRPR agonist has several disadvantages, such as its strong physiological potency and mitogenicity [20]. Furthermore, it was demonstrated that bombesin-based GRPR antagonists have more binding sites than agonists, which should be beneficial for tumor uptake [3,21].

In this study, we report the synthesis of a new PSMA/GRPR-targeting heterodimer that includes a Glu-Ureido PSMA-binding moiety [22] and GRPR antagonist RM26 [23] coupled via a glutamic acid bearing NOTA chelator. The aim of this study was to preclinically evaluate the gallium- and indium-labeled heterodimer in terms of the binding properties to PSMA and GRPR, cellular processing, in vivo targeting, and biodistribution, as well as the imaging properties. 

## 2. Results 

### 2.1. Synthesis of the Heterodimer

The bispecific heterodimeric molecule Glu-Urea-Glu-Aoc-Lys(NOTA)-(PEG)_6_-RM26 (**6**) was synthesized using solid-phase peptide synthesis. The heterodimer was obtained with a 10% yield (based on the initial loading of the resin) and the [M + 2H]^2+^ and [M + 3H]^3+^ species were detected with LC-MS (Figure S1).

### 2.2. Labeling of Heterodimer **6** with Indium-111 and Gallium-68

Heterodimer **6** was successfully labeled with indium-111 and gallium-68, with radiochemical yields exceeding 99% and 98%, respectively. Challenge of the ^111^In-NOTA complex with a 1000-fold molar excess of EDTA demonstrated a high labeling stability, with 0.9 ± 0.6% release in EDTA and 1.2 ± 0.5% release in serum after 1 h incubation. 

### 2.3. In Vitro Specificity 

**Figure 1:**
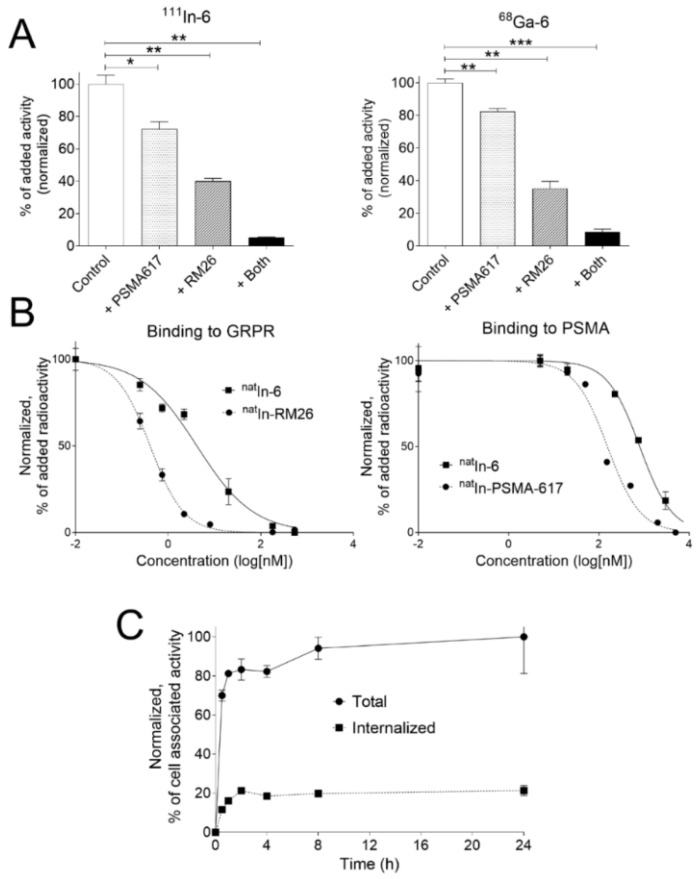
(**A**) Binding specificity of ^111^In-**6** (left) and ^68^Ga-**6** (right) to PC3-PIP cells (prostate-specific membrane antigen (PSMA) positive/gastrin-releasing peptide receptor (GRPR) positive). Cell containing dishes (*n* = 3) were incubated with radiolabeled conjugates (1 nM). One set of dishes was pre-saturated with an excess amount (300 nM) of non-labeled PSMA-617 (+PSMA-617), or RM26 (+RM26), or both of them (+both). Cell-associated activity was normalized to control samples. Data are presented as the average ± standard deviation. (**B**) Binding inhibition of ^111^In-NOTA-PEG_6_-RM26 (left, PSMA was blocked with 1 mM of PSMA-11 in all samples) and ^111^In-PSMA-617 (right, GRPR was blocked with 1 mM solution of RM26 in all samples). Data are presented as the mean value ± standard deviation. (**C**) Cellular processing of ^111^In-**6** on PC3-PIP cells under continuous incubation with 2 nM of labeled heterodimer at 37 °C. Cell-associated activity was normalized to the maximum uptake. Data are presented as the average ± standard deviation.

The binding specificity of ^111^In-**6** and ^68^Ga-**6** was tested on PSMA- and GRPR-positive PC3-PIP cells (Figure 1A), on PSMA-expressing LNCaP cells and GRPR-expressing PC-3 cells (Figure S2). The uptake of ^111^In-**6** and ^68^Ga-**6** on LNCaP and PC-3 cells was significantly reduced when incubated with an excess of corresponding non-labeled targeting agent. The blocking effect was less pronounced in LNCaP cells compared to PC-3 cells, possibly indicating a lower affinity of the heterodimer towards PSMA (Figure S2). For PC3-PIP cells expressing both targets, the binding of labeled conjugates was significantly decreased by the PSMA inhibitor PSMA-617 and by RM26. These data indicate that the binding of the heterodimer is specific to both receptors after labeling (Figure 1A). When the binding of radiolabeled conjugates was simultaneously challenged with PSMA-617 and RM26, the cell-associated activity was significantly lower than for the non-treated cells and for cells pre-incubated with the individual non-labeled binders.

### 2.4. Cellular Processing

Data concerning the cellular processing of ^111^In-**6** by PC3-PIP cells are presented in Figure 1C. Binding of ^111^In-**6** to PC3-PIP cells was rapid, reaching 80% of maximum cell-associated activity after 1 h incubation. The rapid binding was accompanied by slow internalization, with the internalized fraction reaching 21% of cell-associated radioactivity after 24 h of incubation. 

### 2.5. Binding Affinity

The half-maximal inhibitory concentration (IC_50_) was evaluated using ^111^In-PSMA-617 and ^111^In-RM26 as displacement radioligands (Figure 1B). The second molecular target was blocked by the corresponding non-labeled agent. For comparison, the half-maximal inhibitory concentrations of the monomers corresponding to PSMA and GRPR were measured in the same experiment. The combination of the two monomers into heterodimer **6** had a negative effect on the binding properties: IC_50_ values of GRPR decreased 10-fold (from 0.41 ± 0.06 to 4 ± 1 nM) and of PSMA decreased 5-fold (from 160 ± 30 to 824 ± 230 nM).

### 2.6. In Vivo Biodistribution and Targeting Specificity

**Figure 2:**
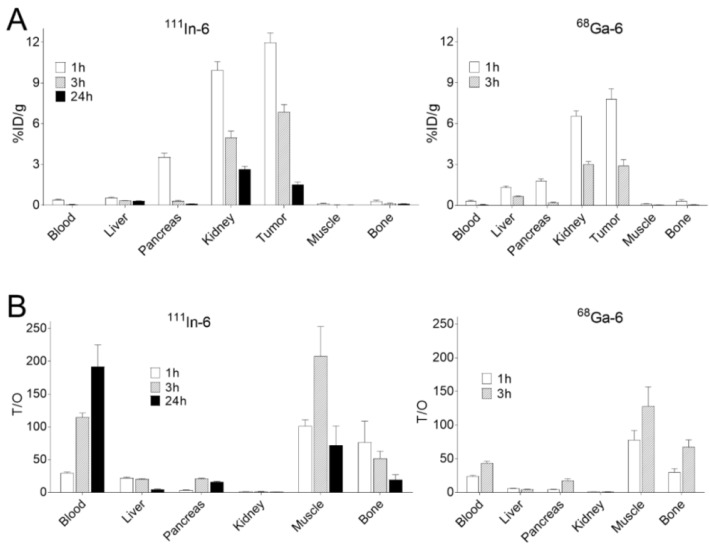
(**A**) In vivo biodistribution of ^111^In-**6** (left) and ^68^Ga-**6** (right) (50 pmol/animal) over time in PC3-PIP-xenografted BALB/c nu/nu mice. Activity uptake in tissues was calculated as the percent of injected dose per tissue weight (%ID/g). (**B**) Tumor-to-normal-tissues ratios for ^111^In-**6** (left) and ^68^Ga-**6** (right). Data are presented as the average ± standard deviation.

The biodistribution and in vivo targeting specificity of radiolabeled heterodimers were studied in mice bearing PC3-PIP tumors in a dual isotope study in which ^111^In-**6** and ^68^Ga-**6** were injected simultaneously (Figure 2A). Both conjugates were rapidly cleared from blood circulation and, at 1 h pi, the activity concentration in blood was below 0.5%ID/g. The excretion of conjugates was predominantly renal, with a low degree of activity re-absorption in the kidneys. Hepatic activity uptake was also low. Elevated activity uptake was found in GRPR-expressing organs (3.5 ± 0.8%ID/g in pancreas, 1.3 ± 0.3%ID/g in stomach, 1 h pi), but not in PSMA-expressing organs (0.3 ± 0.06%ID/g in spleen, 10 ± 2%ID/g in kidneys, 1 h pi). The activity concentration decreased with time in all studied tissues for both conjugates. The biodistribution of ^111^In-**6** and ^68^Ga-**6** was generally similar. However, 1 h pi, ^68^Ga-**6** had a significantly higher uptake in the liver (1.3 ± 0.2%ID/g for ^68^Ga-**6** vs. 0.6 ± 0.1%ID/g for ^111^In-**6**) and a significantly lower uptake in the pancreas (1.8 ± 0.3%ID/g for ^68^Ga-**6** vs. 3.5 ± 0.8%ID/g for ^111^In-**6**).

The tumor activity uptake of ^111^In-**6** and ^68^Ga-**6** was higher compared to all normal tissues at 1 h pi (12 ± 2%ID/g for ^111^In-**6**, 8 ± 2%ID/g for ^68^Ga-**6**) (Figure 2A, Table S1). Although tumor-associated activity decreased with time, tumor uptake remained similar to kidney uptake at all other studied time points; it should be noted that 3 h pi, tumor activity uptake for ^111^In-**6** was significantly higher than that of the kidneys. The clearance of activity from normal tissues was faster compared to that of the tumor, resulting in an overall improvement of tumor-to-nontumor ratios 3 h pi (Figure 2B, Table S2). However, 24 h pi, tumor-to-nontumor ratios for ^111^In-**6** decreased compared to 3 h pi (particularly for the liver, intestine, kidney, muscle, and bone). Tumor-associated activity was significantly higher for ^111^In-**6** than for ^68^Ga-**6** both 1 and 3 h pi. This translated into significantly higher tumor-to-nontumor ratios for ^111^In-**6** for the majority of studied tissues.

**Figure 3:**
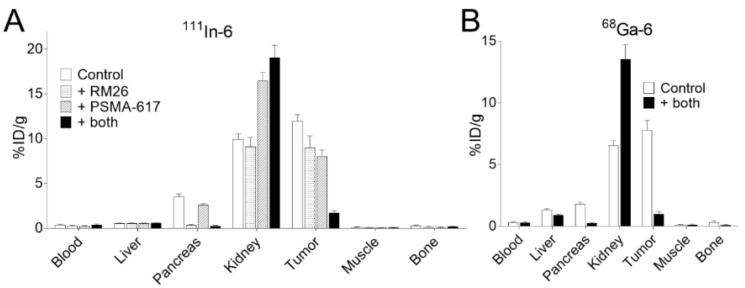
In vivo specificity of ^111^In-**6** (**A**) and ^68^Ga-**6** (**B**) tested in BALB/c nu/nu mice bearing PC3-PIP-xenografts, 1 h pi. Mice were intravenously injected with 50 pmol of ^111^In-**6** or ^68^Ga-**6** alone or together with 1.5 nmol non-labeled RM26, 1.5 nmol non-labeled PSMA-617, or both. Activity uptake in tissues was calculated as the percent of injected dose per tissue weight (%ID/g). Data are presented as the average ± standard deviation.

The in vivo targeting specificity of ^111^In-**6** and ^68^Ga-**6** was evaluated 1 h pi by the co-injection of PSMA- and GRPR-targeting monomers (Figure 3, Tables S3 and S4). The uptake of both radiolabeled conjugates in the tumor, pancreas, and tissues of the GI tract was significantly decreased when co-injected with an excess amount of target specific non-labeled monomers, as well as uptake in the liver and spleen for ^68^Ga-**6**. In an additional experiment, the uptake of ^111^In-**6** in PC3-PIP-xenografted mice was compared with the uptake of ^111^In-**6** co-injected with PSMA- and GRPR-targeting monomers, separately. The co-injection of excess non-labeled GRPR-targeting monomer significantly decreased the uptake in GRPR-expressing organs (*p* < 0.05) and caused a strong tendency to decrease the activity uptake in tumors (*p* = 0.0598), while the addition of the PSMA-targeting monomer significantly decreased the activity uptake in tumors and the pancreas (*p* < 0.05).

### 2.7. Imaging

**Figure 4:**
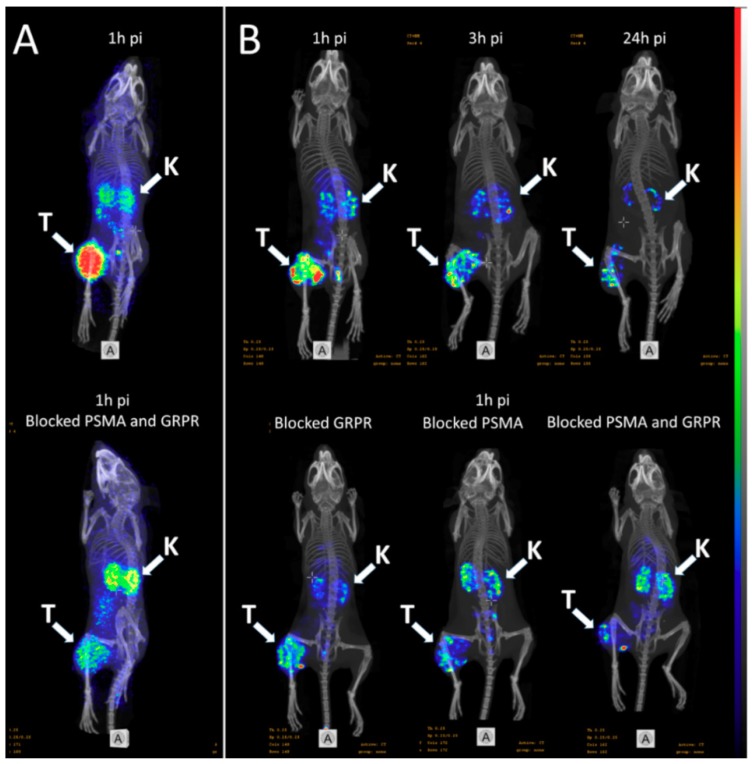
(**A**) micro positron emission tomography (microPET)/CT and (**B**) microSPECT/CT MIP images of PC3-PIP-xenografted mice (PSMA positive/GRPR positive) after an iv injection of ^68^Ga-**6** and ^111^In-**6**. Blocked animals were co-injected with PSMA-617 for the blocking of PSMA and RM26 for the blocking of GRPR, or both.

The imaging of PC3-PIP tumors using microPET/CT for ^68^Ga-**6** (1 h pi) and microSPECT/CT for ^111^In-**6** (1, 3, and 24 h pi) confirmed the ex vivo biodistribution data (Figure 4). Tumors were clearly visualized at all time-points, while weak activity accumulation was only observed in the kidneys among healthy organs. In agreement with ex vivo measurements, imaging contrast improved with time up to 3 h pi, despite the rapid washout of activity from tumors. The superior imaging contrast obtained for ^111^In-**6** compared to ^68^Ga-**6** was also in agreement with the biodistribution data. The imaging of mice after the co-injection of excess PSMA- and GRPR-targeting monomers corroborated with the ex vivo biodistribution pattern: partially decreased activity uptake in tumors, but increased activity uptake in kidneys, were exhibited in the case of PSMA blocking.

## 3. Discussion

The development of imaging agents capable of visualizing both PSMA and GRPR simultaneously could improve the diagnostic accuracy for PCa patients. Both molecular targets are strongly associated with PCa. However, their expression pattern differs, depending on cancer progression. While GRPR expression is substantial in primary PCa (up to 100%) and lymph node metastases (>85%) [11,24], PSMA expression increases with the transition towards androgen independence, and is most associated with high-grade, metastatic disease [25,26]. Diagnostic imaging using agents capable of binding to both of these molecular targets should decrease false negative results and avoid multiple diagnostic imaging procedures. 

**Scheme 1:**
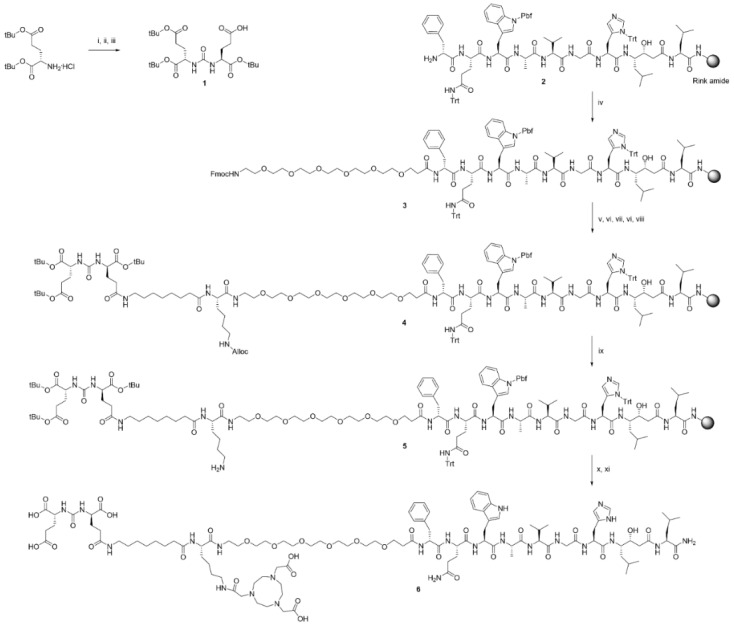
Synthesis of the bispecific heterodimer **6**. (I) triphosgene, TEA, DCM, −78 °C to rt; (II) L-Glu(OBn)-OtBu⋅HCl, TEA, −78 °C to rt; (III) H_2_, Pd/C, MeOH, (IV) Fmoc-NH-dPEG(6)-COOH, PyBOP, DIEA, DMF; (V) Fmoc-Lys(Alloc)-OH, PyBOP, DIEA, DMF; (VI) 20% (*v*/*v*) piperidine in DMF; (VII) Fmoc-8-Aoc-OH, PyBOP, DIEA, DMF; (VIII) 1, PyBOP, DIEA, DMF; (IX) Pd(PPh_3_)_4_, PhSiH_3_, DCM; (X) NOTA-bis(tBu) ester, PyBOP, DIEA, DMF; (XI) TFA, H_2_O, thioanisole, TES.

We have designed and synthesized a short peptide derivative that could bind to PCa-relevant cell surface molecular targets, PSMA and GRPR (Scheme 1). For PSMA targeting, we chose the Glu-Ureido-based PSMA inhibitor DUPA (2-[3-(1, 3-dicarboxy propyl)ureido] pentanedioic acid) and for GRPR targeting, we selected the bombesin-based antagonist RM26 (D-Phe-Gln-Trp-Ala-Val-Gly-His-Sta-Leu-NH_2_ ([D-Phe^6^,Sta^13^,Leu^14^]-bombesin(6−14)). We chose a GRPR antagonist for our molecular design keeping in mind that (i) GRPR antagonists are capable of binding to more receptors than agonists [27] and (ii) GRPR agonists have strong side effects and mitogenicity [20]. The use of an antagonist should increase the activity uptake in tumors and avoid unpleasant side-effects for the patients. Previously, we demonstrated that the introduction of a miniPEG linker at the N terminus of RM26 did not reduce its binding properties to GRPR, but provided rapid blood clearance, a low degree of off-target interaction, and an overall biodistribution profile favorable for the diagnostic imaging of PCa [23]. Conversely, DUPA derivatives with lipophilic linkers should have a better binding affinity to PSMA because they optimally fit the active site and entry tunnel of PSMA [22,28]. For this reason, we introduced the lipophilic (CH_2_)_7_ linker to the PSMA-targeting moiety and hydrophilic PEG_6_ linker to the GRPR-targeting moiety. For labeling function, we chose the NOTA chelator, which provides stable complexes with several radiometals suitable for PET imaging (^68^Ga (T½ = 1 h), ^66^Ga (9.5 h), ^18^F via AlF chemistry (2 h), ^61^Cu (3.4 h), ^64^Cu (12,7 h), and ^55^Co (17.5 h)) and for SPECT imaging (^111^In (67 h)).

Heterodimer **6** was successfully radiolabeled with ^111^In and ^68^Ga with high labeling yields that should allow the purification step to be omitted. This should benefit labeling with short-lived radionuclides. ^111^In-**6** was characterized by a high labeling stability, which is an essential requirement for radiolabeled agents. 

The binding specificity test indicated that the binding of ^111^In-**6** and ^68^Ga-**6** to GRPR- and/or PSMA-expressing cells was receptor-mediated (Figure 1A, Figure S2), suggesting that labeling did not influence the ability of ^111^In-**6** and ^68^Ga-**6** to bind specifically to both targets.

The competitive binding assay revealed a negative effect of dimerization on the binding properties of ^nat^In-**6** towards GRPR and PSMA, with 10-fold higher IC_50_ values for GRPR and 5-fold higher IC_50_ values for PSMA. The observed decrease in the binding capacity could be explained by steric hindrance caused by dimerization. A similar decrease in the binding capacity, especially towards PSMA, was observed for the heterodimers tested by Kopka’s group [17,29]. However, the heterodimer based on naphthyl-containing PSMA-617 did not exhibit a similar tendency [19,30]. High IC_50_ values towards PSMA for the heterodimer and corresponding monomer were not expected in our molecular design: monomeric PSMA inhibitors with similar linkers exhibited IC_50_ values in the low nanomolar range [31]. The moderate affinity of the heterodimer to PSMA can also explain the less pronounced blocking effect of PSMA-617 observed in the in vitro specificity study (Figure 1A, Figure S2).

Cellular activity uptake was rapid, but was not accompanied by internalization (Figure 1C). Slow internalization is expected for the conjugate that has both inhibiting properties towards one target (PSMA) and antagonistic properties towards the other target (GRPR). The slow internalization of the new heterodimer, together with the moderate affinity to PSMA, could lead to a rapid activity wash out from tumors in vivo. However, the high expression of targets in PCa lesions could partially compensate for this by an avidity effect.

The biodistribution of ^111^In-**6** and ^68^Ga-**6** in mice was characterized by a rapid clearance of activity from the blood and normal organs. The excretion of activity was predominantly renal. Interestingly, kidney uptake was very low for the heterodimer tested in this study (on the same level as tumor uptake). For comparison, activity uptake in the kidneys 1 h pi was 3-fold higher than in tumors for the ^99m^Tc-labeled PSMA binder with a similar linker [22], 5-fold higher for ^111^In-PSMA-617 [32], 8-fold higher for ^68^Ga-PSMA-617, and 20-fold higher for ^68^Ga-PSMA-11 [33]. High activity uptake in the kidneys of PSMA-targeting radioagents is expected due to the endogenous expression of PSMA in proximal tubules of normal kidneys [34]. The receptor-mediated nature of renal activity uptake for PSMA binders was previously confirmed by a decrease of activity uptake when the radioagent was co-injected with an excess of non-labeled agent [22,32,33]. Interestingly, in this study, renal activity uptake increased significantly when the PSMA-targeting monomer was co-injected. This apparent contradiction can be explained by the low affinity to PSMA of the tested heterodimer: the concentration of the binder in primary urine increased when a higher agent mass was injected, which led to increased binding to existing receptors in proximal tubules. The data published for the series of PSMA binders with different affinities to PSMA corroborate our results. Kularatne et al. [35] found that the binder with the worst affinity to PSMA (60 nM) had a 10-fold lower activity uptake in kidneys than the binder with a better affinity (14 nM). Elevated activity uptake was found in GRPR-expressing organs (pancreas and GI tissues), but not in PSMA-expressing organs (kidneys and spleen), most likely due to the low affinity of these conjugates towards PSMA. 

Despite the low affinity towards PSMA, the tested heterodimer demonstrated a high activity uptake in tumors 1 h pi: 12 ± 1%ID/g when labeled with ^111^In and 8 ± 2%ID/g when labeled with ^68^Ga. Taking into account that tumor activity uptake could not be blocked with the co-injection of anti-GRPR binder alone, we concluded that the new heterodimer binds to both PSMA and GRPR. Although a direct comparison is not possible, the uptake of ^111^In-**6** and ^68^Ga-**6** in PSMA- and GRPR-expressing xenografts in this study was at least the same or higher compared to the activity uptake in xenografts with either PSMA (LNCaP) or GRPR (PC-3 or AR42J) expression [17,18,29,30]. Tumor activity uptake of ^111^In-**6** dropped by only 25%–30% when the targeted receptors were blocked individually, modeling a situation with PSMA-positive/GRPR-negative and PSMA-negative/GRPR-positive xenografts used in other published studies. These results indicate that ^111^In-**6** and ^68^Ga-**6** should be suitable for the imaging of tumors expressing only one of the receptors. Overall, ^111^In-**6** had a superior biodistribution profile compared to ^68^Ga-**6**, which translated into significantly higher tumor-to-nontumor ratios for ^111^In-**6** for the majority of studied tissues.

The new labeled heterodimer provided high contrast PET and SPECT images of PSMA/GRPR-positive xenografts shortly after administration (1–3 h) due to rapid blood clearance, high tumor uptake, and low uptake in normal tissues and excretory organs, including PSMA-expressing kidneys (Figure 4). In agreement with ex vivo measurements, imaging contrast improved with time up to 3 h pi due to a faster washout of activity from normal tissues compared to the tumor. Despite our expectations that a lipophilic linker would provide a high affinity for the heterodimer towards PSMA, the measured affinity was relatively low. This could explain the rapid activity washout from tumors and the appreciable drop in contrast at a later time point (24 h pi). The imaging of mice co-injected with excess PSMA- or GRPR-targeting monomers revealed the partial blocking of tumor uptake when only one monomer was co-injected and a pronounced blocking effect when both monomers were co-injected. Paradoxically, the low affinity of the new heterodimer to PSMA might improve the imaging properties of this agent by contributing to the low specific renal uptake. However, this phenomenon requires further investigation.

## 4. Materials and Methods 

### 4.1. Synthesis of the Heterodimer

The bispecific heterodimeric molecule Glu-Urea-Glu-Aoc-Lys(NOTA)-(PEG)_6_-RM26 was synthesized by combining the PSMA inhibitor Glu-Urea-Glu and the GRPR binding peptide RM26 via Aoc-Lys(NOTA)-(PEG)_6_ using solid-phase peptide synthesis according to Scheme 1.

All the starting materials and solvents were purchased and used without purification: the amino acids (Novabiochem, Switzerland, Alexis Corporation, Switzerland, Iris Biotech GmbH, Germany or Senn chemicals, Switzerland), HBTU (Novabiochem, Switzerland), PyBOP (Novabiochem, Switzerland), Fmoc-Rink-amide-MBHA (Iris Biotech GmbH, Germany), Fmoc-NH-dPEG(6)-COOH (Iris Biotech GmbH, Germany), NOTA (t-Bu)_2_-OH (CheMatech, France), piperidine (Sigma-Aldrich, Sweden), and DMF (Fisher Scientific, UK). ^1^H and ^13^C NMR spectra were recorded at 25 °C and at 400 and 100 MHz, respectively. Chemical shifts are reported in ppm with the solvent residual peak as the internal standard (CDCl_3_ δ_H_ 7.26, CDCl_3_ δ_C_ 77.16). Analytical electrospray ionization liquid chromatography mass spectrometry (ESI-LC-MS) analysis was performed on a Dionex Ultimate 3000 with a Bruker amaZon iontrap mass spectrometer using a Phenomenex Kinetex core-shell C18 (4.6 × 50 mm) 2.6 μm column and a 1.5 mL/min, acetonitrile/water (0.05% formic acid) gradient with detection in positive mode.

Compound **1** ((S)-5-(tert-butoxy)-4-(3-((S)-1,5-di-tert-butoxy-1,5-dioxopentan-2-yl)ureido) -5-oxopentanoic acid) was synthesized according to a literature procedure, with minor modifications [22]. Triethylamine (418 µL, 3.0 mmol) was added to a solution of L-Glu(O*t*Bu)-O*t*Bu HCl (443 mg, 1.5 mmol) and triphosgene (148 mg, 0.5 mmol) in dry DCM (10 mL) at −78 °C. After stirring for 20 min at −78 °C, the reaction mixture was allowed to reach room temperature and was stirred for 1 h. The reaction mixture was cooled to −78 °C and a solution of L-Glu(OBn)-O*t*Bu HCl (528 mg, 1.6 mmol) in DCM (3 mL) was added, followed by triethylamine (307 µL, 2.2 mmol). The reaction mixture was allowed to reach room temperature and reacted overnight. KHSO_4_ (1 M) and DCM were added and the layers were separated. The aqueous layer was extracted with DCM and the combined organic fractions was washed with brine and dried over Na_2_SO_4_. The crude product (773 mg) was dissolved in 12 mL MeOH and 10% Pd/C (77 mg) was added. The reaction mixture was hydrogenated for 14 h and thereafter filtered through a pad of Celite and washed with DCM. The material obtained after evaporation was purified by flash chromatography (gradient iso-hexane:EtOAc 4:1 to 1:1) to give **1** in 212 mg (30%). ^1^H NMR (CDCl_3_): δ 5.99 (d, 1H, NH), 5.50 (d, 1H, NH), 4.45 (m, 1H, CH), 4.31 (m, 1H, CH), 2.23–2.48 (m, 4H, 2 × CH_2_), 2.02–2.19 (m, 2H, CH_2_), 1.75–1.95 (m, 2H, CH_2_), 1.47 (s, 9H, 3 × CH_3_), 1.44 (s, 9H, 3 × CH_3_), 1.42 (s, 9 H, 3 × CH_3_). ^13^C NMR (CDCl_3_): δ 175.9, 173.2, 172.4, 171.7, 158.0, 82.7, 82.1, 80.6, 53.5, 53.0, 31.5, 30.5, 28.5, 28.05, 28.00, 27.96, 27.8. LC-MS (M+H^+^): 489.3.

H_2_N-[D-Phe^6^, Sta^13^, Leu^14^]Bombesin [6,7,8,9,10,11,12,13,14], and H_2_N-PEG_6_-RM26, **2**, in Scheme 1, were synthesized on a 100 µmolar scale by solid-phase peptide synthesis (SPPS) on Rink Amide 4-methylbenzhydrylamine resin (0.1 mmol, loading 0.53 mmol/g) (Merck, Darmstadt) in a 2 mL disposable syringe equipped with a porous polyethylene filter using standard Fmoc/*t*-Bu conditions, as described earlier [21]. For the Fmoc-amino acids, the following side-chain-protecting groups were used: His (Trt), Trp (Boc), and Gln (Trt). Fmoc-statine was used without side-chain protection. Additionally, 20% Piperidine in DMF (4 × 2 mL) was used to remove the Fmoc group.

To obtain the protected Glu-Urea-Glu-Aoc-Lys(Alloc)-(PEG)_6_-RM26, **6**, Fmoc-NH- dPEG(6)COOH (3 equiv. 300 µmol) was coupled in DMF (2 mL) overnight in the presence of DIEA (4.5 equiv. 450 µmol). The resin was washed extensively with DMF, DCM, and MeOH and dried in vacuum. In total, 50 µmol of the peptide on resin was further coupled to Fmoc-Lys(Alloc)-OH (5 equiv, 250 µmol), the Fmoc group was removed, the resin was washed with DMF, and Fmoc-8-Aoc-OH (4 equiv, 200 µmol) was coupled for 2 h with PyBOP (4 equiv, 200 µmol) in the presence of DIEA (8 equiv, 400 µmol). After deprotection and washing steps, the previously synthesized compound **1** (2 equiv, 100 µmol) was coupled overnight using PyBOP (2 equiv, 100 µmol) and DIEA (4 equiv, 200 µmol) to obtain **4**. It was then washed and a part of the dried peptide was transferred to a reaction tube, and Alloc de-protection was performed, as explained previously [36]. In brief, the vacuum-dried Alloc-protected compound **4** (31 mg, about 7.5 µmol), PhSiH_3_ (20 equiv.), and Pd(PPh_3_)_4_ (0.25 equiv.) were reacted in 2 mL DCM under nitrogen. The reaction was rotated for 2 h and the resin was transferred to a syringe equipped with a porous polyethylene filter and washed extensively with DCM and DMF. The partly protected peptide, **5**, was finally coupled to the NOTA-bis(tBu) ester (2,1 equiv, 16 µmol) in DMF with PyBOP (2,1 equiv, 16 µmol) as the coupling reagent and DIEA (4 equiv, 32 µmol) as the base. After extensive washing with DMF, DCM, and MeOH, the resin was dried in vacuum to yield 28 mg partly-protected peptide.

A solution of TFA/thioanisol/water (90/6/4, 2 mL) was added to the resin (24 mg) and the suspension was rotated at room temperature for 1.5 h. Triethylsilane (50 µL) was added and the resin was removed by filtration and rinsed with TFA (3 × 300 µL). The filtrate was concentrated in a stream of nitrogen and the crude product was precipitated by the addition of ether (12 mL). The precipitate was recovered by centrifugation, washed with ether (3 × 7 mL), and dried in vacuum overnight to yield 10.6 mg of the crude product. The material was dissolved in 25% aq. MeCN (2 mL) and purified with preparative reversed high-performance liquid chromatography (RP-HPLC) on a Nucleodur HTech 58m C18 column (125 × 21 mm) using a CH_3_CN/H_2_O gradient with 0.1% trifluoroacetic acid (TFA) at a flow rate of 10 mL/min and with UV detection at 220 nm.

Lyophilization of the appropriate fractions from the HPLC purification afforded 2.6 mg of purified product **6**, corresponding to a total yield of 10% (based on the initial loading of the resin).

### 4.2. Characterization of the Heterodimer

The purified peptide **6** was characterized with LC-MS and the purity was confirmed with analytical HPLC. LC-MS [M+2H]^2+^: 1153.6 and [M+3H]^3+^: 769.1. Analytical RP-HPLC on a Nucleodur C18 HTec 5 µm column (50 × 4.6 mm) using a CH_3_CN/H_2_O gradient with 0.1% trifluoroacetic acid (TFA) at a flow rate of 2 mL/min and with UV detection at 220 nm showed 95% purity (Figure S1).

### 4.3. Labeling of Heterodimer 6 with Indium-111 and Gallium-68

Buffers for radiolabeling were prepared using high-quality Milli-Q water (resistance >18 MΩ cm) and pretreated with Chelex 100 resin (Bio-Rad Laboratories, Richmond, USA) to remove metal contaminants. The majority of chemicals used were purchased from Sigma-Aldrich.

For ^111^In labeling, an aqueous solution of 10 nmol (10 µL in Milli-Q water) of **6** was buffered with 80 µL (0.2 M, pH 5.5) of ammonium acetate (Merck). After the addition of 20–40 MBq (50–100 µL in 0.05 M hydrochloric acid) of ^111^In (Mallinckrodt Radiopharmaceuticals Sverige AB), the reaction mixture was incubated for 30 min at 80 °C. To evaluate the labeling stability, ^111^In-**6** was incubated in serum for 1 h at 37 °C, or in the presence of a 1000-fold molar excess of EDTA disodium salt (Sigma) for 1 h at RT. The labeling yield and in vitro stability were analyzed using instant thin-layer chromatography (ITLC) strips (Agilent Technologies) eluted with citric acid (0.2 M, pH 2.0).

For ^68^Ga labeling, a ^68^Ge/^68^Ga generator (Cyclotron Co. Obninsk, Russia) was eluted with 0.1 M HCl (800 µL/min, Merck) in fractions of 800 µL. The second fraction was used for labeling. The aqueous solution of heterodimer (10 nmol, 10 μL in Milli-Q water) was buffered with sodium acetate (0.2 M, pH 3.9, 200 μL), followed by the addition of ^68^Ga (200 µL, 200 MBq). The mixture was incubated at 80 °C for 10 min. The labeling yield was analyzed by ITLC.

### 4.4. In Vitro Experiments

In vitro characterization of the radiolabeled heterodimer was performed using the isogenic human prostate adenocarcinoma cell line PC3-PIP (PSMA- and GRPR-positive, obtained from Dr. Warren Heston, Cleveland Clinic). Cells were cultured in RPMI-1640 media supplemented with 10% fetal calf serum (Sigma), PEST (penicillin 100 IU/mL, streptomycin 100 g/mL), and 2 mM L-glutamine (all from Biochrom AG, Berlin, Germany). This medium is referred to as complete medium in the text. Approximately every second of passaging, 10 µg/mL puromycin was added to the previously described medium in order to maintain the selection pressure. Trypsin-EDTA (0.05% trypsin, 0.02% EDTA in buffer) was purchased from Biochrom AG. A change of media was done twice a week, and the cells were seeded two days prior to experiments.

#### 4.4.1. In Vitro Specificity Test for ^111^In-**6** and ^68^Ga-6 on PC3-PIP Cells

The in vitro specificity of ^111^In-**6** and ^68^Ga-**6** was evaluated on PC3-PIP cells. The cells were incubated with 1 nM of ^111^In-**6** or ^68^Ga-**6** solutions for 1 h at 37 °C. For blocking, for each radio-conjugate, three sets of dishes were pre-incubated with a 300-fold excess of unlabeled PSMA-617, unlabeled NOTA-PEG_6_-RM26, or a combination of both. The blocking solutions were added 10 min prior to the addition of the radiolabeled compounds. After being washed once with serum-free media, cells were treated with 0.5 mL trypsin solution. After cell detachment, measurements of cell-associated activity were performed against standards in an automated gamma well counter (3-inch NaI(Tl) detector, 2480 Wizard2, PerkinElmer). Additionally, an in vitro binding specificity assay was performed using PC-3 (specificity to GRPR) and LNCaP (specificity to PSMA) (cell lines were purchased from ATCC, USA).

#### 4.4.2. Cellular Processing

For determining the cellular processing and internalization, PC3-PIP cells were incubated with 2 nM of ^111^In-**6** at 37 °C. At predetermined time points (0.5, 1, 2, 4, 8, and 24 h after the start of incubation), the incubation medium was discarded and cells were washed with serum-free medium, followed by 2 mL acid buffer (4 M urea in 0.2 M glycine buffer, pH 2.0). The acid wash solution was collected in vials and the radioactivity of the samples was measured using an automated gamma-counter. These samples were considered to represent the membrane-bound fraction. Subsequently, a base solution (NaOH, 1 M, 2 mL) was added to the cells, followed by incubation for 30 min at 37 °C. The base solution was collected and the radioactivity of these samples was considered to reflect the internalized fraction.

#### 4.4.3. Binding Affinity

The half-maximal inhibitory concentration (IC_50_) values towards PSMA and GRPR were determined for ^nat^In-**6** using PC3-PIP cells. ^111^In-PSMA-617 was used as a displacement radioligand for PSMA and ^111^In-DOTA-PEG_2_-RM26 was used for GRPR, respectively. Due to the presence of both targets on PC3-PIP cells, in each experiment, the secondary target was blocked with either 1 mM solution of NOTA-PEG_6_-RM26 or 2 mM PSMA-11. For comparison, the IC_50_ values were determined for the corresponding monomers towards PSMA and GRPR under the same experimental conditions. Briefly, cell monolayers were incubated with the radioligand (1 nM) at 4 °C for 5 h in the presence of increasing concentrations of ^nat^In-loaded conjugates (0–540 nM for GRPR-targeting analogs, and 0–7500 nM for PSMA-targeting, respectively). Following incubation, cells were collected and the cell-associated radioactivity was measured in an automated gamma-counter. The IC_50_ values were calculated by nonlinear regression using GraphPad Prism software (GraphPad Software Inc., San Diego, CA, USA).

### 4.5. In Vivo Biodistribution and Targeting Specificity

All animal experiments were planned and performed in accordance with the Swedish national legislation on the protection of laboratory animals and the study plans were approved by the Ethics Committee for Animal Research in Uppsala. Euthanasia was performed by the intraperitoneal injection of a ketamine-xylazine (Ketalar-Rompun) solution (200 mg/kg ketamine/Ketalar and 20 mg/kg xylazine/Rompun), and all efforts were made to minimize suffering. Prior to implantation, female outbred BALB/c nu/nu mice (*n* = 30) were quarantined for 1 week. The average animal weight at the time of the experiment was 16 ± 1 g. Xenografts expressing both PSMA and GRPR were established by the subcutaneous injection of ~0.8 × 10^7^ PC3-PIP cells/mouse. Tumors were grown for 7 days and the average tumor weight at the time of the experiment was 0.3 ± 0.1 g. Before the experiments, animals were randomized into groups of four. Blood, salivary glands, lung, liver, spleen, pancreas, stomach, small intestines, kidneys, muscle, bone, and tumor samples were collected for radioactivity measurements. The gastrointestinal tract with content and the rest of the carcass were also collected. The activity of samples was measured together with the injection standard.

To evaluate the biodistribution over time, three groups of mice were intravenously co-injected through the tail vein with ^111^In-**6** and ^68^Ga-**6** (300 kBq for ^68^Ga-**6** and 40 kBq for ^111^In-**6**) and sacrificed 1, 3, and 24 h pi. The total injected peptide mass was adjusted to 50 pmol/mouse.

The specificity of in vivo targeting was evaluated for both ^111^In-**6** and ^68^Ga-**6** at 1 h pi. For the ^111^In study, in addition to the injection of ^111^In-**6** (40 kBq, 50 pmol/mouse) in all mice, three out of four groups of mice were co-injected with an excess amount of either non-labeled PSMA-617 (1.5 nmol), non-labeled NOTA-PEG_6_-RM26 (1.5 nmol), or a combination of both. The ^68^Ga study was performed similarly, with two groups of mice injected with ^68^Ga-**6** (300 kBq, 50 pmol/mouse). One of the two groups was co-injected with a combination of non-labeled PSMA-617 (1.5 nmol) and non-labeled NOTA-PEG_6_-RM26 (1.5 nmol).

### 4.6. Imaging

Whole body SPECT/CT scans of the mice bearing PC3-PIP xenografts injected with ^111^In-**6** (400 kBq, 100 pmol/mouse) were performed using nanoScan SPECT/CT (Mediso Medical Imaging Systems, Hungary) at 1, 3, and 24 h pi. Additionally, three mice were co-injected with either non-labeled PSMA-617 (1.5 nmol), non-labeled NOTA-PEG_6_-RM26 (1.5 nmol), or a combination of both, and imaged 1 h pi. CT scans were acquired at the following parameters: 50 keV, 670 μA, 480 projections, and 5 min acquisition time. SPECT scans were carried out using ^111^In energy windows (154 keV–188 keV; 221 keV–270 keV), a 256  ×  256 matrix, and a 30 min acquisition time. The CT raw data were reconstructed using Nucline 2.03 Software (Mediso Medical Imaging Systems, Hungary). SPECT raw data were reconstructed using Tera-Tomo™ 3D SPECT.

PET/CT scans of the mice injected with ^68^Ga-**6** (1.8 MBq, 100 pmol/mouse) were performed using nanoScan PET/MRI (Mediso Medical Imaging Systems, Hungary) at 1 h pi. To evaluate the in vivo specificity, one mouse was co-injected with a combination of non-labeled PSMA-617 (1.5 nmol) and non-labeled NOTA-PEG_6_-RM26 (1.5 nmol). CT acquisitions were performed as previously described using nanoScan SPECT/CT immediately after PET acquisition employing the same bed position. PET scans were performed for 30 min. PET data were reconstructed into a static image using the Tera-Tomo™ 3D reconstruction engine. CT data were reconstructed using Filter Back Projection. PET and CT files were fused and analyzed using Nucline 2.03 Software.

## 5. Conclusions

Considering the high degree of heterogeneity among prostate tumors, it is unlikely that PSMA- or GRPR-targeting agents alone will be ideal in all clinical situations. The lack of PSMA or GRPR expression in highly heterogeneous lesions may result in failure to notice these lesions using either PSMA- or GRPR-targeting agents alone and could lead to false-negative findings. This could have a significant influence on the choice of therapeutic strategies. Therefore, targeting both PSMA and GRPR with an anti-GRPR/PSMA heterodimer may have a high clinical impact in the diagnostic imaging and therapy of prostate cancer.

In conclusion, the new anti-GRPR/PSMA heterodimer NOTA-DUPA-RM26 labeled with galium-68 (for PET) and indium-111 (for SPECT) is a suitable candidate for the imaging of GRPR and PSMA expression in PCa shortly after administration. Further investigations of the effect of the molecular design on the structure–properties relationship for the heterodimer are required to optimize its composition.

## Supplementary Materials

The following are available online at https://www.mdpi.com/2072-6694/11/9/1371/s1: Figure S1: Characterization of heterodimer **6**, Glu-Urea-Glu-Aoc-Lys(NOTA)-(PEG)6-RM26. Analytical RP-HPLC chromatogram (a) and MS spectrum (b) of purified bispecific heterodimeric molecule **6**; Figure S2: In vitro specificity test for ^111^In-**6** and ^68^Ga-**6** on PC3 (GRPR positive) and LNCaP (PSMA positive cells); Table S1: In vivo biodistribution of ^111^In-**6** and ^68^Ga-**6** (50 pmol/animal) over time in PC3-PIP-xenografted BALB/c nu/nu mice; Table S2: Tumor-to-organ ratios of ^111^In-**6** and ^68^Ga-**6** tested in BALB/c nu/nu mice bearing PC3-PIP-xenografts, 1, 3, and 24 h pi for ^111^In-**6** and 1 and 3 h pi for ^68^Ga-**6**; Table S3: In vivo specificity of ^111^In-6 and ^68^Ga-6 tested in BALB/c nu/nu mice bearing PC3-PIP-xenografts, 1 h pi; Table S4: Tumor-to-organ ratios of ^111^In-**6** and ^68^Ga-**6** tested in BALB/c nu/nu mice bearing PC3-PIP-xenografts, 1 h pi.
