# Supplementary material for: Bispecific GRPR-Antagonistic Anti-PSMA/GRPR Heterodimer for PET and SPECT Diagnostic Imaging of Prostate Cancer
Source: Cancers (Basel). 2019 Sep 14;11(9):1371. doi: 10.3390/cancers11091371 (PMC6771040; doi:10.3390/cancers11091371)
Supplement: Supplementary file 1 [file cancers-11-01371-s001.pdf]

Supplementary Materials

Bispecific GRPR-antagonistic anti-PSMA/GRPR heterodimer for PET and SPECT diagnostic imaging of prostate cancer

Bogdan Mitran, Zohreh Varasteh, Ayman Abouzayed, Sara S. Rinne, Emmi Puuvuori, Maria De Rosa, Mats Larhed, Vladimir Tolmachev, Anna Orlova and Ulrika Rosenström

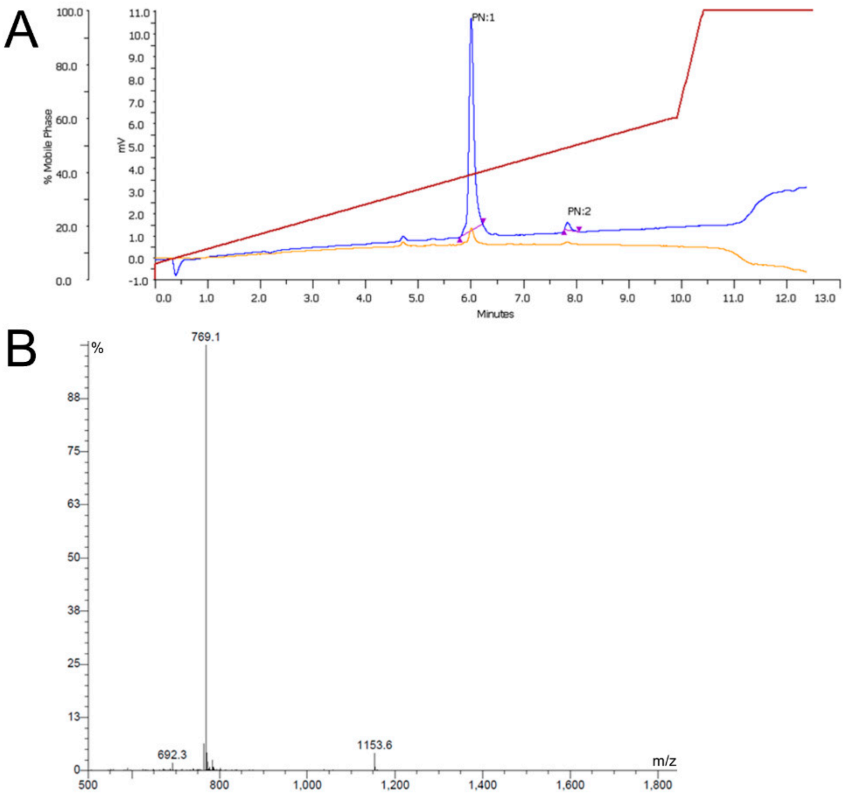

Figure S1. Characterization of heterodimer **6**, Glu-Urea-Glu-Aoc-Lys(NOTA)-(PEG)$_6$-RM26. Analytical RP-HPLC chromatogram (**a**) and MS spectrum (**b**) of purified bispecific heterodimeric molecule **6**.

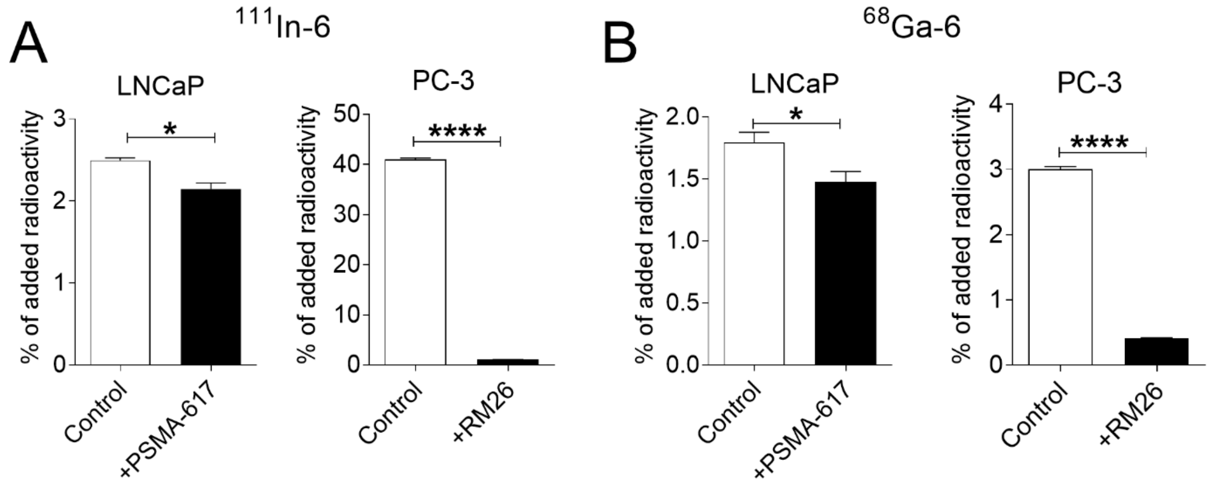

Figure S2. Binding specificity of ^{111}In-**6** (**a**) and ^{68}Ga-**6** (**b**) to LNCaP cells (PSMA positive) and PC-3 (GRPR positive). Cell containing dishes (n=3) were incubated with radiolabeled conjugates (1 nM). One set of dishes was pre-saturated with excess amount (300 nM) of non-labeled PSMA-617 (+ PSMA-617), or RM26 (+ RM26). Data are presented as average ± standard deviation.

Table S1. In vivo biodistribution of ^{111}In-**6** and ^{68}Ga-**6** (50 pmol/animal) over time in PC3-PIP-xenografted BALB/c nu/nu mice. Activity uptake in tissues was calculated as percent injected dose per tissue weight (%ID/g). Data are presented as average ± standard deviation.

	^{111}In-**6**			^{68}Ga-**6**	
Organ	1h	3h	24h	1h	3h
Blood	0.4±0.1 [a,b]	0.06±0.02	0.009±0.005 [a]	0.33±0.09 [a]	0.07±0.01
Salivary glands	0.18±0.07 [a,b]	0.07±0.02	0.06±0.02	0.19±0.08 [a]	0.07±0.02
Lung	0.39±0.09 [a,b]	0.13±0.06	0.046±0.008 [a]	0.6±0.2 [a]	0.12±0.06
Liver	0.6±0.1 [a,b,c]	0.34±0.02 [c]	0.31±0.05	1.3±0.2 [a]	0.67±0.06
Spleen	0.28±0.06 [a,b,c]	0.12±0.03 [c]	0.06±0.04 [a]	0.49±0.06 [a]	0.28±0.09
Pancreas	3.5±0.8 [a,b,c]	0.33±0.06	0.10±0.04 [a]	1.8±0.3 [a]	0.2±0.1
Stomach	1.3±0.3 [a,b,c]	0.26±0.09	0.04±0.03 [a]	0.8±0.2 [a]	0.1±0.1
Small intestine	1.2±0.5 [a,b]	0.17±0.04	0.055±0.003 [a]	0.8±0.3 [a]	0.13±0.08
Kidneys	10±2 [a,b,c]	4.9±0.9 [c]	2.6±0.4 [a]	6.6±0.8 [a]	3.0±0.4
Tumor	12±2 [a,b,c]	7±1 [c]	1.5±0.4 [a]	8±2 [a]	2.9±0.9
Muscle	0.13±0.05 [a,b]	0.04±0.01	0.03±0.02	0.11±0.05 [a]	0.03±0.01
Bone	0.3±0.2	0.14±0.05 [c]	0.10±0.05	0.3±0.2	0.05±0.03
GI tract	0.8±0.3	0.6±0.4	0.2±0.1	0.5±0.2	0.4±0.2
Carcass	2.5±0.7 [a,b]	0.9±0.1 [c]	0.44±0.06 [a]	1.9±0.6 [a]	0.6±0.1

The organ uptake values are expressed as a percentage of injected dose per gram of tissue weight (%ID/g) except for carcass and GI tract, for which the values are expressed as a percentage of the injected dose per sample (%ID). Significant difference (p<0.5) at the same time point.

a. *Conjugate significantly differs from the 3h pi time-point*
b. *Conjugate significantly differs from the 24h pi time-point*
c. *Conjugate significantly differs from ^{68}Ga-**6** at the same time-point*

Table S2. Tumor-to-organ ratios of ^{111}In-**6** and ^{68}Ga-**6** tested in BALB/c nu/nu mice bearing PC3-PIP-xenografts, 1, 3, 24 h pi for ^{111}In-**6** and 1, 3 h pi for ^{68}Ga-**6**. Mice were intravenously injected with 50 pmol of ^{111}In-**6** or ^{68}Ga-**6**. Data are presented as average ± standard deviation.

	^{111}In-**6**			^{68}Ga-**6**	
Organ	1h	3h	24h	1h	3h
Blood	30±4	115±13	192±66	24±3	44±6
Salivary glands	72±13	93±10	26±4	43±8	43±8
Lung	32±3	64±30	34±11	15±3	28±17
Liver	22±4	20±2	4.8±0.8	5.9±0.9	4±1
Spleen	44±6	59±16	30±16	16±4	11±1
Pancreas	3.5±0.8	21±1	16±2	4.4±0.6	18±6
Stomach	10±3	29±12	61±52	10±4	45±39
Small intestine	12±4	42±9	27±6	11±3	33±27
Kidneys	1.2±0.2	1.4±0.2	0.6±0.1	1.2±0.1	0.9±0.2
Muscle	101±25	210±90	72±59	78±28	128±57
Bone	75±50	52±22	19±15	30±11	67±21

Table S3. In vivo specificity of ^{111}In-**6** and ^{68}Ga-**6** tested in BALB/c nu/nu mice bearing PC3-PIP-xenografts, 1 h pi. Mice were intravenously injected with 50 pmol of ^{111}In-**6** or ^{68}Ga-**6** alone or together with 1.5 nmol non-labeled RM26, or 1.5 nmol non-labeled PSMA-617, or both. Activity uptake in tissues was calculated as percent injected dose per tissue weight (%ID/g). Data are presented as average ± standard deviation.

	^{111}In-**6**				^{68}Ga-**6**	
Organ	Control	+RM26	+PSMA-617	+Both	Control	+Both
Blood	0.4±0.1	0.3±0.1	0.29±0.02	0.4±0.2	0.33±0.09	0.3±0.2
Salivary glands	0.18±0.07	0.14±0.05	0.12±0.04	0.16±0.06	0.19±0.08	0.11±0.06
Lung	0.39±0.09	0.31±0.09	0.4±0.1	0.6±0.3	0.6±0.2	0.7±0.2
Liver	0.6±0.1	0.55±0.06	0.54±0.07	0.58±0.06	1.3±0.2	0.9±0.1[a]
Spleen	0.28±0.06	0.17±0.03[a]	0.20±0.04	0.23±0.07	0.49±0.06	0.28±0.06[a]
Pancreas	3.5±0.8	0.3±0.1[a]	2.6±0.2	0.28±0.09[a]	1.8±0.3	0.3±0.1[a]
Stomach	1.3±0.3	0.17±0.05[a]	0.88±0.09[a]	0.3±0.2[a]	0.8±0.2	0.3±0.1[a]
Small intestine	1.2±0.5	0.19±0.04[a]	0.7±0.2	0.24±0.07[a]	0.8±0.3	0.3±0.1[a]
Kidneys	10±2	9±2	16±2[a]	19±3[a]	6.6±0.8	14±2[a]
Tumor	12±2	9±2	8±1[a]	1.7±0.6[a]	8±2	0.9±0.4[a]
Muscle	0.13±0.05	0.06±0.02	0.07±0.03	0.10±0.03	0.11±0.05	0.1±0.1
Bone	0.3±0.2	0.15±0.09	0.12±0.03	0.19±0.08	0.3±0.2	0.11±0.08
GI tract	0.8±0.3	0.4±0.2	0.7±0.1	0.7±0.4	0.5±0.2	0.6±0.5
Carcass	2.5±0.7	1.7±0.7	1.9±0.5	5±4	1.9±0.6	4±4

The organ uptake values are expressed as a percentage of injected dose per gram of tissue weight (%ID/g) except for carcass and GI tract, for which the values are expressed as a percentage of the injected dose per sample (%ID). Significant difference ($p<0.5$) at the same time point.

d. Conjugate significantly differs from control

Table S4. Tumor-to-organ ratios of ^{111}In-**6** and ^{68}Ga-**6** tested in BALB/c nu/nu mice bearing PC3-PIP-xenografts, 1 h pi. Mice were intravenously injected with 50 pmol of ^{111}In-**6** or ^{68}Ga-**6** alone or together with 1.5 nmol non-labeled RM26, or 1.5 nmol non-labeled PSMA-617, or both. Data are presented as average ± standard deviation.

	^{111}In-**6**				^{68}Ga-**6**	
Organ	Control	+RM26	+PSMA-617	+Both	Control	+Both
Blood	30±4	37±14	28±3	4.5±0.7	24±3	3.3±0.5
Salivary glands	72±13	67±9	74±35	11±1	43±8	10±4
Lung	32±3	30±2	24±4	3.1±0.6	15±3	1.4±0.5
Liver	22±4	16±3	15±0.8	2.9±0.8	5.9±0.9	1.1±0.5
Spleen	44±6	54±6	41±11	8±3	16±4	4±1
Pancreas	3.5±0.8	27±3	3.0±0.3	6±3	4.4±0.6	5±3
Stomach	10±3	52±3	9±2	6±4	10±4	4±2
Small intestine	12±4	48±8	12±5	8±4	11±3	4±3
Kidneys	1.2±0.2	0.9±0.1	0.49±0.06	0.09±0.02	1.2±0.1	0.07±0.02
Muscle	101±25	151±27	132±34	17±3	78±28	11±6
Bone	75±50	73±31	69±26	10±6	30±11	11±7
